# Injectable Nanocomposite Hydrogels of Gelatin-Hyaluronic Acid Reinforced with Hybrid Lysozyme Nanofibrils-Gold Nanoparticles for the Regeneration of Damaged Myocardium

**DOI:** 10.1021/acsami.3c03874

**Published:** 2023-05-18

**Authors:** Tiago Carvalho, Raquel Bártolo, Sónia N. Pedro, Bruno F. A. Valente, Ricardo J. B. Pinto, Carla Vilela, Mohammad-Ali Shahbazi, Hélder A. Santos, Carmen S. R. Freire

**Affiliations:** † CICECO − Aveiro Institute of Materials, Department of Chemistry, University of Aveiro, Campus de Santiago, 3810-193 Aveiro, Portugal; ‡ Department of Biomedical Engineering, University Medical Center Groningen, University of Groningen, Ant. Deusinglaan 1, 9713 AV Groningen, The Netherlands; § W.J. Kolff Institute for Biomedical Engineering and Materials Science, University Medical Center Groningen, University of Groningen, Ant. Deusinglaan 1, 9713 AV Groningen, The Netherlands; ∥ Drug Research Program, Division of Pharmaceutical Chemistry and Technology, Faculty of Pharmacy, University of Helsinki, FI-00014 Helsinki, Finland

**Keywords:** biopolymeric composites, gold nanoparticles, injectable hydrogels, lysozyme nanofibrils, myocardium regeneration

## Abstract

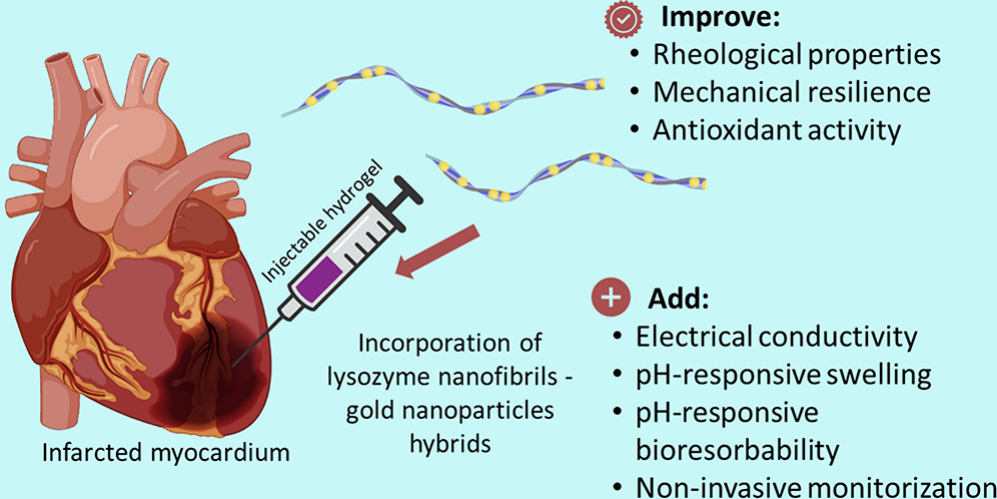

Biopolymeric injectable hydrogels are promising biomaterials for myocardial regeneration applications. Besides being biocompatible, they adjust themselves, perfectly fitting the surrounding tissue. However, due to their nature, biopolymeric hydrogels usually lack desirable functionalities, such as antioxidant activity and electrical conductivity, and in some cases, mechanical performance. Protein nanofibrils (NFs), such as lysozyme nanofibrils (LNFs), are proteic nanostructures with excellent mechanical performance and antioxidant activity, which can work as nanotemplates to produce metallic nanoparticles. Here, gold nanoparticles (AuNPs) were synthesized in situ in the presence of LNFs, and the obtained hybrid AuNPs@LNFs were incorporated into gelatin-hyaluronic acid (HA) hydrogels for myocardial regeneration applications. The resulting nanocomposite hydrogels showed improved rheological properties, mechanical resilience, antioxidant activity, and electrical conductivity, especially for the hydrogels containing AuNPs@LNFs. The swelling and bioresorbability ratios of these hydrogels are favorably adjusted at lower pH levels, which correspond to the ones in inflamed tissues. These improvements were observed while maintaining important properties, namely, injectability, biocompatibility, and the ability to release a model drug. Additionally, the presence of AuNPs allowed the hydrogels to be monitorable through computer tomography. This work demonstrates that LNFs and AuNPs@LNFs are excellent functional nanostructures to formulate injectable biopolymeric nanocomposite hydrogels for myocardial regeneration applications.

## 1 Introduction

In the past few years, there has been a growing interest in purely using biopolymers to design and prepare scaffolds with improved properties and functionalities for myocardial regeneration applications.^1^ Among these scaffolds, injectable hydrogels, which are hydrogels that can be easily extruded through a thin syringe needle using the strength of an average person, stand out due to their minimally invasive nature, moldability to the applied zone, and potential multifunctional performance.^2^ When designing a hydrogel for regenerative medicine, biopolymers, such as polysaccharides and proteins, are usually preferred, mainly due to their biocompatibility, hydrophilicity, and bioresorbability.^3^ For example, gelatin, collagen, HA, chitosan, and decellularized extracellular matrix (dECM), have been extensively explored to prepare hydrogels for this application.^4^

As a result of its ubiquitous occurrence in the ECM, and being an immunomodulatory biopolymer,^5^ HA is a widely used biopolymer for myocardial regeneration applications.^6^ HA is a nonsulfated glycosaminoglycan composed of repeating disaccharides of β-1,4-d-glucuronic acid and β-1,3-N-acetyl-d-glucosamine, and interacts with other ECM biopolymers through covalent and noncovalent interactions.^7^ Besides being an ECM component, HA promotes cell adhesion, migration, and morphogenesis.^8^ It has also been reported that HA endorses cell proliferation and differentiation, helping in the development and modeling of inflamed tissue.^7^ This biopolymer can form hydrogels through covalent crosslinking;^7,8^ however, similarly to other natural hydrogels, it lacks some functional properties, such as electrical conductivity and appropriate antioxidant activity.^4^ Nonetheless, in combination with other polymers, HA has been used to prepare hydrogels for myocardial regeneration applications. For instance, injectable hydrogels of HA-chitosan/β-glycerophosphate^9^ and oxidized HA-hydrazided HA containing poly(lactic-co-glycolic acid) microparticles^10^ were loaded with mesenchymal stem cells and revealed good mechanical compliance. When tested in vivo, they promoted cardiac function, angiogenesis and reduced cardiomyocyte apoptosis. In a different study, a cell-free injectable hydrogel composed of methacrylate HA, reactive oxygen species (ROS)-cleavable hyperbranched polymers, and O_2_-generating catalase functioned as a ROS scavenging and O_2_-producing material.^11^ Additionally, the in vivo tests revealed the improvement of cardiac function inhibition, reduction of cell apoptosis, angiogenesis promotion, and reduction of infarcted area.

Another widely used biopolymer for the stated application is gelatin, which in addition to mimicking the EMC, also provides feasibility for cell attachment and growth.^12^ Being a derivative of the partial hydrolysis of collagen, which easily forms hydrogels, it is widely used in the development of injectable hydrogels for myocardial regeneration.^4^ To add desired properties not found on this biopolymer^13^ while still making use of its biological benefits, gelatin is usually blended with synthetic polymers (*e.g.*, poly(N-isopropylacrylamide)^14^) or chemically modified (*e.g.*, gelatin methacrylate^15^) to prepare injectable hydrogels more suited for the regeneration of infarcted myocardial tissue.

Unfortunately, to date, no hydrogel formulation has displayed the required biological, mechanical, and functional properties for an adequate transition for clinical trials.^4^ Exploring underutilized biopolymeric nanostructures, such as protein amyloid NFs, might reveal a promising strategy to answer this problem.^16^ Protein NFs are nanostructures that are formed from the stacking of β-sheet-misfolded proteins or peptides,^17^ which can occur naturally^18^ or be produced in vitro.^19,20^ These stacks result in highly organized quaternary structures stabilized by hydrogen bonds, inducing a rigid internal order to the fibrils.^21^ Typically, these structures present a morphology of unbranched filaments helically twisted along their axis.^22^ As a result of their morphology, biological nature, and unique attributes, these NFs possess remarkable mechanical properties, thermal stability, and insolubility in aqueous media.^23^ Additionally, amyloid-β fibrils have been shown to trigger minimal inflammatory responses, being quickly phagocyted as macrophages make contact with them.^24^ Furthermore, it was shown that synthetic amyloid-β fibrils do not trigger any cellular transcriptional response.^25^ For these characteristics, in a previous work,^16^ LNFs were added as functional nanofillers to gelatin electrospun patches. Their incorporation into the patches improved their mechanical performance, bioresorbability ratio, and antioxidant activity. This latter improvement, antioxidant activity, is of very high interest for the intended application for the hydrogels studied in this work. Due to the abnormal increase of ROS post-infarct, which further damages the surrounding tissue, it is of the most importance to neutralize them as soon as possible with antioxidant agents.^26^ Also, due to their unique properties and biological features, protein NFs have been mostly explored for potential biomedical applications, such as tissue regeneration, biosensors, drug delivery systems, and bioelectronics.^16,21^ Another interesting feature of protein NFs is their ability to act as nanotemplates and stabilizers to produce metallic nanoparticles, such as AuNPs, in particular, to create chain structures of these nanostructures.^27^ AuNPs have also been used for myocardial regeneration applications^28^ due to their functionalities and applications, including imaging,^29,30^ nanobiosensing,^28,30^ drug release,^30^ electrical conductivity,^28^ and diagnosis and therapy applications.^31^

Cardiovascular diseases, with myocardial infarction as the most common disorder,^2,32^ are the current leading cause of death worldwide, representing 32% of all global deaths in 2019.^33^ As a result of the lack of regeneration capacity of the myocardial tissue, after an infarction episode, adaptative and pathologic pathways are triggered.^34^ Usually, after the accumulation of ROS, rupture of the ECM, accompanied by disturbance of the tissue mechanical properties, heart deformation, and failure normally occur.^35^ Considering that heart transplantation is still the only efficient treatment, alternative approaches to address the high mortality associated with cardiovascular diseases are of utmost importance.^35,36^ Taking this scenario into account, as well as the need for low-invasiveness methodologies, as previously stated, injectable hydrogels have emerged as innovative materials to be administrated into the infarcted site.^4^ Additionally, injectable hydrogels can also incorporate drugs, biomolecules, and/or cells to either stimulate a desirable response or simply promote the formation of new tissue that could replace the damaged one.^4^

Taking altogether, the present study aims to design an injectable, biocompatible hydrogel with the ability to recreate the microenvironment and the mechanical compliance found in healthy myocardium tissue. Specifically, the LNFs were functionalized with AuNPs by in situ synthesis and incorporated into gelatin-HA injectable hydrogels. These nanocomposite hydrogels were prepared with fixed quantities of gelatin and HA and different amounts of functionalized LNFs (or pure LNFs for comparison purposes). All hydrogels were characterized in terms of their mechanical and rheological properties, injectability, conductivity, antioxidant activity, bioresorbability, cytotoxicity, and ability to incorporate and release a model drug (CHIR 99021 trihydrochloride) to assess their suitability for the envisioned application.

## 2 Experimental Section

### 2.1 Chemicals and Cell Line

Acetic acid (≥99.7%, Sigma-Aldrich), CellTiter-Glo reagent (Promega Corporation), CHIR 99021 trihydrochloride (Toronto Research Chemicals, Canada), choline chloride (≥98%, Sigma-Aldrich), Dulbecco’s modified Eagle’s medium (DMEM, HyClone), 2,2-diphenyl-1-picrylhydrazyl (DPPH, Aldrich), gelatin from porcine skin (Sigma-Aldrich), glycine (≥98.5%, Sigma-Aldrich), gold (III) chloride trihydrate (≥99.9%, Sigma-Aldrich), heat-inactivated fetal bovine serum (HIFBS, HyClone), hen egg white lysozyme (∼70000 U mg^–1^, Sigma-Aldrich), HA (≥1.8 MDa, Bloomage Biotechnology Corporation Limited, China), l-glutamine (EuroClone SpA), orthophosphoric acid (Merck, Germany), osmium tetroxide (99.95%, Electron Microscopy Sciences), poly(ethylene glycol) diglycidyl ether (PEGDGE, Aldrich), penicillin–streptomycin (EuroClone SpA, Italy), phosphate-buffered saline (PBS, Sigma-Aldrich), nonessential amino acids (NEAA, HyClone), sodium borohydride (95%, BDH Chemicals, UAE), sodium pyruvate (Gibco), and Triton X-100 (Merck, Germany). Other chemicals and solvents were of laboratory grade. Rat cardiomyoblast (H9c2, 2-1) cells were obtained from ATCC CRL1446 (USA).

### 2.2 LNFs Preparation

LNFs were prepared according to the procedure reported by Silva et al.,^20^ with minor modifications. Briefly, 160 mg of lysozyme were placed into a Falcon tube and set aside. In another Falcon tube, a deep eutectic solvent (DES) was prepared by mixing 1.4 g of cholinium chloride and 0.6 mL of acetic acid. Then, 38 mL of a solution prepared with Milli-Q water containing 0.2% (v/v) of HCl and 0.15% (m/v) of glycine were added to the Falcon tube containing the DES and homogenized. Afterward, this solution was used to dissolve the lysozyme, followed by incubation overnight at 70 °C, with stirring. The obtained suspension of LNFs was centrifuged at 5000 rpm for 45 min at 4 °C, and the supernatant was removed. The LNFs were resuspended in Milli-Q water and dialyzed using a dialysis tubing with a cutoff of 12–14 kDa (Shodex, Germany) for 3 days, changing the outer Milli-Q water every 24 h.

### 2.3 In Situ AuNPs Synthesis

The AuNPs were prepared in situ in the presence of the LNFs, following conditions previously described.^37^ In detail, the suspension of LFNs was concentrated through centrifugation, and its consistency was determined. Then, sufficient amount of this suspension to obtain 160 mg of LNFs was added to a Falcon tube. Then, 30 mL of a stock solution of HAuCl_4_ (5.0 mM) was added to the Falcon tube containing the LNFs. After the suspension was homogenized, four aliquots of 290 μL of NaBH_4_ (0.074 M) were slowly added to the suspension, vigorously stirring the suspension after each addition. After leaving the suspension to react for 30 min, it was transferred into a dialysis tubing with a cutoff of 10 kDa and dialyzed for 3 days, changing the outer Milli-Q water every 24 h. Finally, to determine the quantity of gold, aliquots of LNFs were freeze-dried, digested with aqua regia, and analyzed through Inductively coupled plasma optical emission spectrometry using a Horiba Jobin Yvon spectrometer (USA).

### 2.4 Hydrogels Preparation

A stock of Milli-Q water with pH adjusted to 12.0 with NaOH 1.0 M was used to prepare all solutions and suspensions used for the hydrogels production. A stock solution of HA 1.5% (w/v) was prepared using Milli-Q water and set aside for later use. The pH of the LNF and AuNPs@LNF suspensions was also adjusted to 12.0 and diluted to obtain a LNF concentration of 0.55% (w/v). To prepare 20 mL of each hydrogel, 0.55 g of gelatin was weighed into five different Falcon tubes. Then, to obtain the five different hydrogels, the gelatin was dissolved as follows: in one tube, it was dissolved in 10 mL of Milli-Q water; in two tubes, the gelatin was dissolved in 5 mL of Milli-Q water and either in 5 mL of LNF suspension or AuNPs@LNF suspension; and in the last two tubes, it was dissolved either in 10 mL of LNF suspension or AuNPs@LNF suspension. Afterward, 10 mL of HA was added to each of the five prepared Falcon tubes. After homogenization, the pH was checked and adjusted to 12.0 if necessary, and 500 μL of PEGDGE was added slowly to each of the Falcon tubes and vigorously stirred to ensure the uniform dissolution of the crosslinker. After crosslinking for 48 h at room temperature, the hydrogels were stored at 4 °C for later use.

### 2.5 Attenuated Total Reflection-Fourier Transform Infrared (ATR-FTIR) Spectroscopy

ATR-FTIR spectra of the freeze-dried hydrogels and pure biopolymers were obtained on a PerkinElmer spectrometer equipped with a single horizontal Golden Gate ATR cell. For each sample, 64 scans were recorded between 4000 and 500 cm^–1^, with a resolution of 2 cm^–1^, in the transmission mode.

### 2.6 Injectability

Injectability was evaluated using a mechanical testing machine (Instron 5966 (USA) instrument with Bluehill 3 software) in the compression mode and using a 50 N load cell, as previously described by Chen et al.^38^ In summary, the hydrogels were loaded into 1 mL syringes capped with needles with different gauges (G) and lengths (1/2 inch length: 26, 27, and 30 G; 3/4-inch length: 27 G). Each syringe was vertically fixed and held in a container placed on the mechanical machine bottom anvil, with the needle tip submerged in PBS. The injection force was measured using a top 57 mm anvil, with a flow rate of 2 mL h^–1^, for 2 min.

### 2.7 Rheological Studies

Dynamic rheological tests of the hydrogels, before and after crosslinking, were done on a Kinexus Pro rheometer (Malvern Instruments Limited, Malvern, UK). All hydrogel samples in the form of 20 mm diameter disks were placed in the equipment. Time-sweep oscillatory tests of the hydrogels were performed at 1 Hz frequency, with a 1.0 mm gap for the uncrosslinked hydrogels and a 2.0 mm gap for the crosslinked counterparts, for the duration of 600 s. The frequency-sweep experiments were performed at shear rates ranging from 0.1 to 100 Hz. All measurements were performed at 37 °C.

### 2.8 Cyclic Compressive Stress

The cyclic compressive stress–strain measurements were performed on hydrogel samples, as previously described,^15^ using a universal mechanical testing machine (Instron 3343 instrument with Bluehill software) in the compression mode and using a 50 N load cell. The hydrogel samples were prepared as previously described but inside 24 mm diameter wells to obtain well-defined 5 mm thick cylinder-shaped samples. A cyclic compressive strain rate of 50.0 mm min^–1^ was employed, and the strain level was set to release at 30%. Each measurement was composed of 50 compress and release cycles.

### 2.9 Conductivity

Prior to the conductivity measurements, the injectable hydrogels contained in the Falcon tubes were warmed up in a water bath at 37 °C. The conductivity was directly measured by immersing a probe InLab 731 (Mettler Toledo) into the hydrogels. Triplicates were used to measure the conductivity of all different hydrogels.

### 2.10 Antioxidant Activity

The antioxidant activity of the injectable hydrogels was assessed by the DPPH radical scavenging assay. A stock solution of DPPH was prepared by dissolving 20 mg of DPPH in 50 mL of methanol. Samples of 150 mg of each hydrogel were immersed, in triplicate, in vials containing 3.75 mL of methanol. The samples were left overnight to reach solvent equilibrium between the water inside the hydrogel and the added methanol. A vial simply containing 3.75 mL of methanol was used as the control. After adding 0.25 mL of the stock solution to each prepared vial, they were incubated at 37 °C in an orbital shaker at 100 rpm. The absorbance of the samples (Abs._sample_) was measured at 517 nm on a Shimadzu UV-1800 spectrophotometer (Japan) at 0.25, 0.5, 1, 2, 3, 4, 8, 18, and 24 h by pipetting the aliquots into a quartz cuvette. The antioxidant activity of each sample was calculated according to eq 1
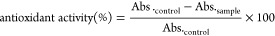
1

### 2.11 Swelling Ratio

The swelling ratio of the prepared hydrogels was determined by measuring their weight variation. In detail, the hydrogels were weighed (W_0_), immersed in PBS solutions (pH 6.0 and 7.4), and incubated at 37 °C in an orbital shaker at 100 rpm, for 0, 1, 2, 4, 8, 16, 24, and 48 h. At each time point, the hydrogels were removed from the PBS solutions, the excess of buffer was cleared off using filter papers, and reweighed (*W*_s_). The swelling ratio was calculated according to eq 2
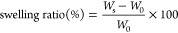
2

### 2.12 Scanning Electron Microscopy (SEM) Imaging

SEM micrographs of the hydrogels before and after swelling for 24 h were obtained using a Hitachi SU-70 microscope (Japan) operating at 15 kV. The samples were prepared by freeze-drying the hydrogels after immersing them in liquid nitrogen. Before SEM imaging, all samples were coated with carbon. The pore areas on each hydrogel sample were measured using the image processing software ImageJ.

### 2.13 Bioresorbability of the Hydrogels

For each hydrogel, specimens with around 100 mg were weighed and incubated in PBS buffers (pH 6.0 and pH 7.4) at 37 °C for different periods (0, 1, 3, 7, 14, 21, and 28 days) in an orbital mixer at 100 rpm. The buffers were carefully changed every day with a fresh one. For each time point, samples were removed from the buffer solution, washed with Milli-Q water, frozen, lyophilized, and reweighed to determine the amount of mass lost during the incubation by comparing with the initial lyophilized mass. At least three replicates were performed for each sample and time point.

### 2.14 Evaluation of the Cytotoxicity of the Hydrogels

To evaluate the cytotoxicity of the injectable hydrogels toward H9c2 cells, the cell viability was accessed for 1, 3, 7, 14, and 21 days. Cell culturing was performed in 96-well plates. The wells to be evaluated were seeded with 5000 H9c2 cells. The cells were incubated with DMEM with 10% (v/v) HIFBS, 1% (w/v) NEAA, 1% (w/v) L-glutamine, penicillin (100 IU mL^–1^), and streptomycin (100 mg mL^–1^). After seeding, each plate was incubated at 37 °C, with an atmosphere of 95% relative humidity and 5% CO_2_, until reaching a desired time point. Afterward, the medium was carefully discarded from each well, and the plate was washed twice with HBSS (pH 7.4). Then, 50 μL of CellTiter-Glo and 50 μL of HBSS were added to each well. After wrapping the plate with aluminum foil, incubation for 30 min with mild agitation in an orbital shaker was carried out. The cell viability was measured with a Varioskan Flash spectral scanning multimode reader (Thermo Fisher Scientific Inc.). The calculations were based on comparisons with the positive control wells, using the corresponding blank wells as background values.

### 2.15 Ex Vivo Computer Tomography

A freshly harvested pig heart was bought at a local slaughterhouse. Its myocardium was cut into pieces of 3 cm length, 1.5 cm width, and 1 cm height. Using a 27 G needle, 50 μL of the hydrogel containing AuNPs@LNFs were injected 0.5 cm deep into a piece of the myocardium. Afterward, each piece was analyzed using a PerkinElmer IVIS Lumina II instrument. The excitation filter was set to 640 nm, the emission filter to Cy5.5, and the readings had a duration of 1 s.

### 2.16 Drug Release Studies

For the drug release studies, the hydrogels were prepared in the same fashion as the ones previously described, with the addition of 200 μg of a model drug, CHIR 99021 trihydrochloride, to each mL of hydrogel suspension, before crosslinking. Hydrogel samples of around 2.0 mL were incubated in triplicates, in flasks containing PBS with pH 6.0, in an orbital mixer at 37 °C, with an agitation speed of 100 rpm. At each time point, an aliquot of 500 μL was collected from each container and substituted with fresh PBS. Then, each aliquot was filtered through a 0.45 μm nylon syringe filter and injected into a high-performance liquid chromatography-diode-array detector (HPLC–DAD, Shimadzu Prominence, Japan) for drug quantification. The HPLC analyses were performed with an analytical C18 reversed-phase column (250 mm × 4.60 mm), Kinetex 5 μm C18 100 Å (Phenomenex). The mobile phase consisted of 50% (v/v) of acetonitrile and 50% (v/v) of ultrapure water with 0.3% (v/v) of orthophosphoric acid, with its pH adjusted to 6.0.^39^ The separation was conducted in isocratic mode, at a flow rate of 0.8 mL min^–1^ and using an injection volume of 10 μL. The column oven and the autosampler operated at 37 °C. The wavelength was set at 274 nm.^39^ The drug amount measured on each aliquot was calculated through its absorbance against a calibration curve. Afterward, the released quantity of the drug at a time point was calculated using eq 3, where *m*_drug_aliquots_ is the total drug mass removed from the aliquots taken from a flask until the time point, *C*_measured_ is the drug concentration in the aliquot taken on that time point, *V* is the total volume inside the flask, and *m*_T_ is the total mass of the drug loaded into the sample.
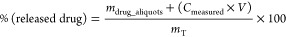
3

### 2.17 Statistical Analysis

The results are expressed as mean ± standard deviation (SD) of at least three independent sets of measurements. Statistical analysis was done using a one-way analysis of variance with the level of significance set at probabilities of *p < 0.05, **p < 0.01, and ***p < 0.001, analyzed with OriginPro9.0 software (OriginLab Corp.).

## 3 Results and Discussion

**Figure 1:**
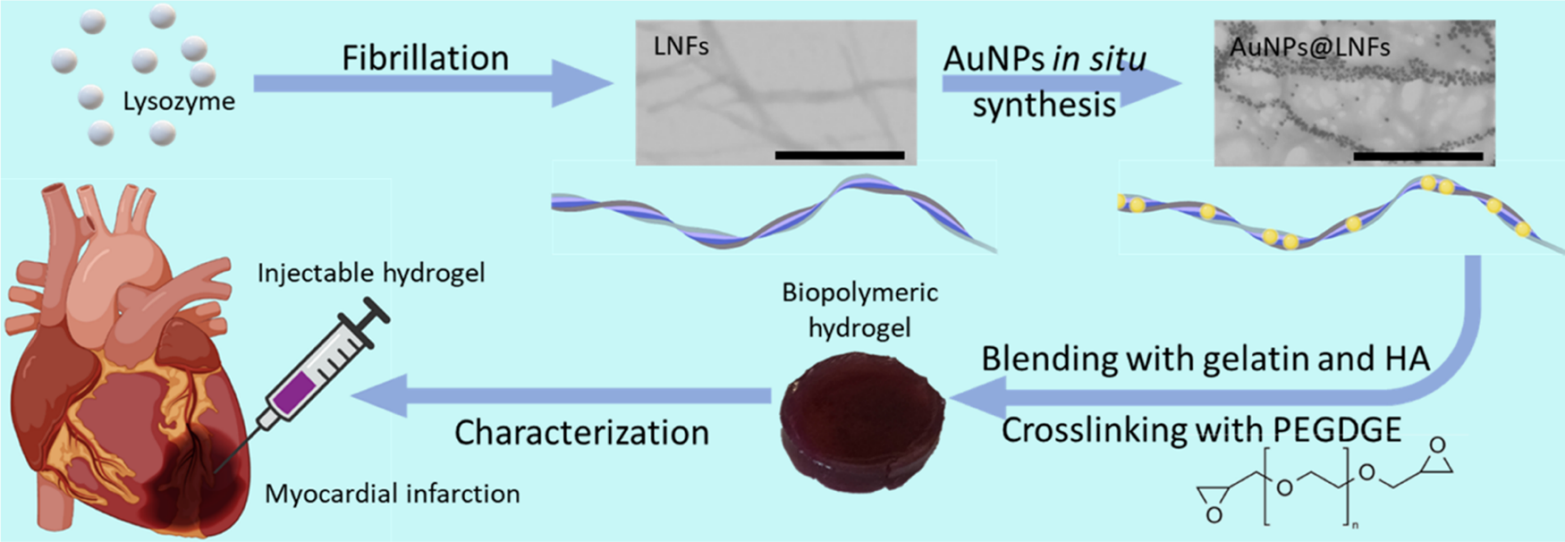
Schematic diagram displaying the preparation of an injectable hydrogel containing LNFs loaded with AuNPs, gelatin, and HA, crosslinked with PEGDGE. Characterizations were performed considering myocardial regeneration applications. Scale bars, 500 nm.

Injectable nanocomposite hydrogels with improved properties and distinct functionalities, namely, resistance to mechanical stress, electrical conductivity, antioxidant activity, pH-responsive swelling, and bioresorbability, were prepared. LNFs were exploited as functional nanofillers and templates to incorporate AuNPs into the hydrogels. Gelatin and HA, together with these hybrid nanostructures, were combined to prepare biopolymeric hydrogels (Figure 1) with appropriate structural and biological properties.^4^ LNFs with a thickness of around 17.9 ± 2.1 nm were obtained by fibrillation of lysozyme from hen egg white, using a DES.^20^ The spherical AuNPs, prepared in situ in the presence of LNFs, have a diameter of 5.8 ± 2.0 nm and correspond to 14.3 ± 0.6% of the total mass of the AuNPs@LNFs hybrid, as determined by inductively coupled plasma optical emission spectrometry. This particle size is adequate for the intended application since it was previously determined that AuNPs in the size range of 5 nm do not affect the integrity of cardiac muscle cells.^28^ Regarding these nonbiodegradable NPs excretion, after being released from the hydrogel, Poon et al.,^40^ demonstrated that once in the bloodstream, the ones smaller than 5.5 nm (almost half of the ones obtained here) will be excreted through urine. Meanwhile, the ones bigger than 5.5 nm will become retained long-term within the Kupffer cells. Despite the toxicity of AuNPs caused by their accumulation still being a controversial theme,^41^ considering the present work, they can be tailored to be smaller than 5.5 nm, becoming totally excretable once released from the hydrogel.

Five different hydrogels were prepared (Table 1), each containing the same amounts of gelatin and HA but increasing contents of LNFs with or without AuNPs functionalization. The crosslinking of the hydrogels was achieved through a reaction with PEGDGE. The hydrogels containing only gelatin and HA are transparent, while the incorporation of pure LNFs turned the hydrogels opaquer. Those with AuNPs@LNFs have a red to dark red color, typical of AuNPs.^42^

**Table 1:** Identification and Composition of the Different Injectable Hydrogels

name	[gelatin] %(w/v)	[HA] %(w/v)	[LNFs] %(w/v)	[AuNPs] %(w/v)
GHA-0LNFs	2.75	0.75	0.00	0.00
GHA-1LNFs	2.75	0.75	0.14	0.00
GHA-2LNFs	2.75	0.75	0.28	0.00
GHA-1AuNPs@LNFs	2.75	0.75	0.14	0.02
GHA-2AuNPs@LNFs	2.75	0.75	0.28	0.04

All nanocomposite hydrogels were then characterized to confirm the crosslinking and in terms of their injectability, rheological performance, mechanical properties, antioxidant activity, conductivity, swelling ratio, bioresorbability, *in vitro* citotoxicity toward H9c2 cells, and their ability to incorporate and release a model drug (CHIR 99021 trihydrochloride), to assess their potential for myocardial regeneration applications.

### 3.1 Structural and Mechanical Characterization

**Figure 2:**
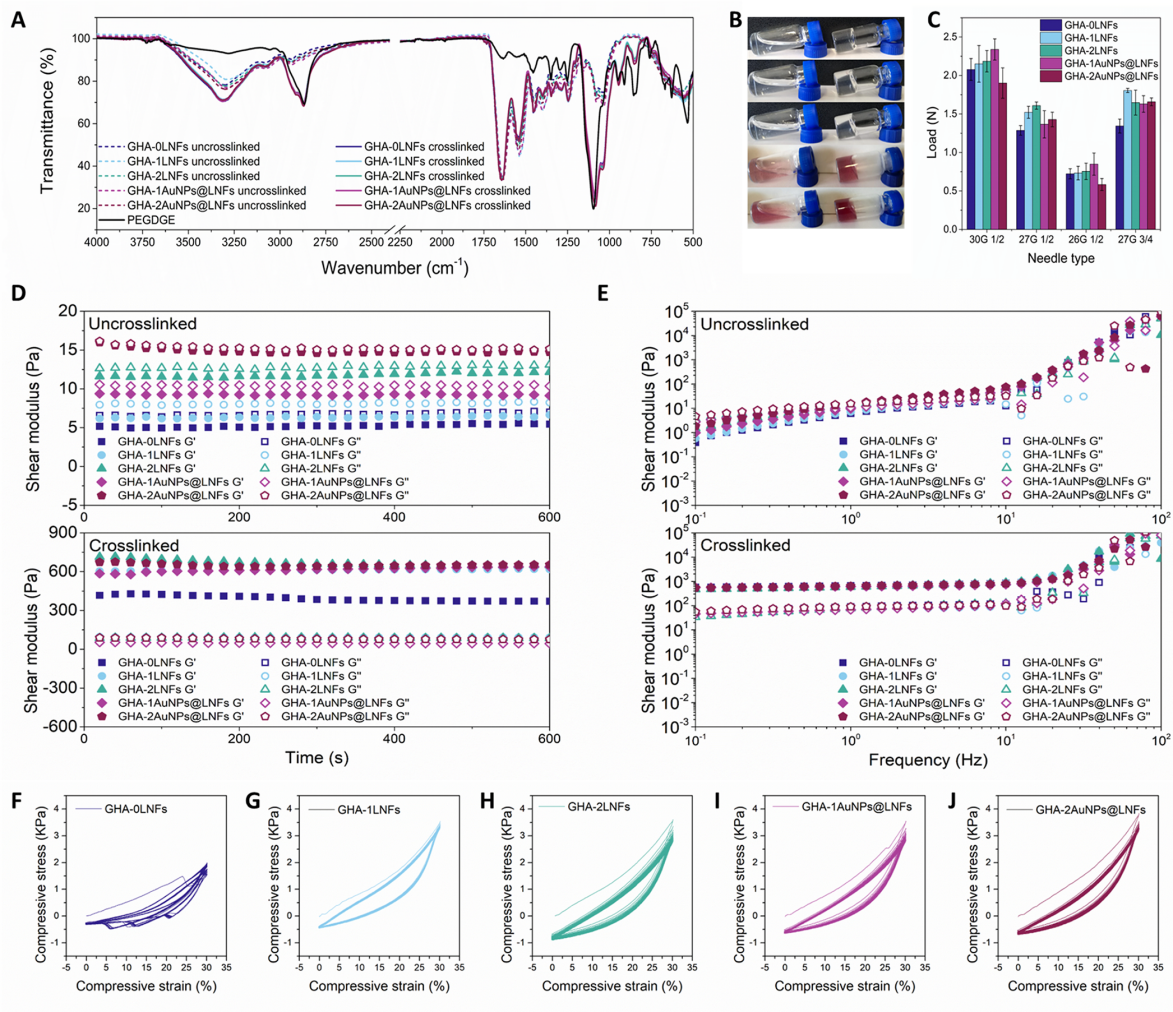
(A) ATR-FTIR spectra of the prepared hydrogels before (dashed lines) and after the addition of PEGDGE and crosslinking for 48 h (solid lines) and PEGDGE (solid black line). (B) Appearance of the hydrogels before and after being crosslinked with PEGDGE (left and right pictures, respectively), when immersed in a water bath at 60 °C. (C) Graphical display of the maximum load measured while extruding the hydrogels through the different syringe needles (30 G 1/2 inch, 27 G 1/2 inch, 26 G 1/2 inch, and 27 G 3/4 inches). (D) Hydrogels’ rheological properties over 10 min at 1 Hz before and after crosslinking (top and bottom, respectively). *G*′ represents the storage modulus, and *G*″ represents the loss modulus. (E) Hydrogels’ viscosity with frequencies ranging from 0.1 to 100 Hz before and after crosslinking (top and bottom, respectively). (F–J) Cyclical compressive tests of all prepared hydrogels (GHA-0LNFs, GHA-1LNFs, GHA-2LNFs, GHA-1AuNPs@LNFs, and GHA-2AuNPs@LNFs, respectively) for 50 cycles.

ATR-FTIR spectroscopy of the lyophilized hydrogels was performed to confirm their chemical structure, particularly the crosslinking reaction of the used biopolymers with PEGDGE (structure shown in Figure 1). Comparing the spectra plotted in Figure 2A, before and after adding PEGDGE, some variations on several absorption bands are revealed, which can be separated into two groups: from PEGDGE itself, as it is not present in the noncrosslinked hydrogels, and from the crosslinking reaction between the epoxy terminal groups of PEGDGE and the nucleophilic lateral groups of the biopolymers.^43^ For the PEGDGE, the vibrations around 1100 and 950 cm^–1^ are attributed to C–O–C stretching vibrations, around 2910 and 2850 cm^–1^ to C–H stretching of methylene and at around 850 cm^–1^ to C–H bending vibration.^43,44^ The observed increase in the intensity of the band around 3400 cm^–1^ and the decrease of the PEGDGE band at 950 cm^–1^ after the reaction allows us to confirm the crosslinking between the biopolymers and the PEGDGE.^43^ Additionally, a quick experiment was performed on the hydrogels before and after crosslinking them with PEGDGE. Hydrogels contained in a vial were immersed in a water bath at 60 °C for 30 s. Then, it was observed if they kept their shape, as demonstrated in Figure 2B, easily confirming the crosslinking reaction success.

Afterward, the force needed to extrude the hydrogels through syringe needles with different diameters (30, 27, and 26 G) and lengths (1/2 and 3/4 inches) was measured. Considering that a human thumb can exert a force of up to 40 N,^45^ the results presented in Figure 2C and Video Clip S1 show that all of the prepared hydrogels are easily injectable since the maximum load needed was under 2.5 N. Additionally, it was observed that the addition of LNFs and AuNPs@LNFs did not impact the force required to extrude the hydrogels. As expected, thinner needles (higher G) with the same length require a higher pressure for a hydrogel to be extruded through them (around 2.5, 1.5, and 0.75 N, for the needles with 30, 27, and 26 G, respectively). The same behavior was observed when comparing needles with different lengths (1/2 and 3/4 inches, both with 27 G). The load was verified to be around 1.50 N when using the shorter needle and slightly increase to 1.75 N when using the longer one.

To characterize their viscoelastic properties, two different rheological assessments were performed on the prepared hydrogels at 37 °C before and after crosslinking. One was performed at a constant shear frequency of 1 Hz over a period of 10 min (Figure 2D), while the other was performed with an increasing shear frequency ranging from 0.1 to 100 Hz (Figure 2E). The storage modulus or elastic modulus (*G*′) relates to the elastic energy stored in the hydrogels, which allows hydrogels reversibility after suffering deformation, describing the solid-state behavior of the samples. Meanwhile, the loss modulus or viscous modulus (*G*″) indicates the amount of stored energy associated with irreversible deformation, describing the liquid-state behavior of the sample.^15^ Regarding the first rheological assessment (Figure 2D) before hydrogel crosslinking *G*″ is higher than *G*′ for all hydrogels, which is characteristic of viscoelastic liquids since there are no strong bonds between the individual biopolymer macromolecules.^46^ The addition of LNFs or AuNPS@LNFs to the formulations did not alter this gap difference. Then, after adding PEGDGE to the hydrogels, *G*′ becomes higher than *G*″ for all hydrogels, due to the formation of bonds inside the material, confirming the crosslinking reaction.^47^ The gap between *G*′ and *G*″ is higher on the hydrogels containing LNFs than the ones not containing them. This phenomenon should be due to the higher total concentration of biopolymers in the hydrogels containing LNFs. Regarding the second rheological assessment (Figure 2E), the noncrosslinked hydrogels reveal stability up to a shear frequency of 10 Hz. Most importantly, the crosslinked hydrogels have their *G*′ higher than their *G*″ with a shear frequency up to 100 Hz, which indicates the hydrogels’ stability and good mechanical properties.^48^ Additionally, before reaching the frequency of 100 Hz, there was an increase of both *G*′ and *G*″, a profile similar to what was observed on other gelatin:HA-based hydrogels,^49^ as well as on collagen-based and Matrigel hydrogels,^50^ indicating the existence of noncovalent interactions in the hydrogel network, in addition to the covalent ones formed during crosslinking.^49^ These behaviors observed during both rheological assessments were also reported on studies about other injectable hydrogels for myocardial regeneration applications, such as a gelatin methacrylate-oxidized dextran-based hydrogel,^46^ and an acrylate-modified polycarboxybetaine-dithiothreitol-based hydrogel.^51^ The incorporation of AuNPs did not affect the rheological properties of the hydrogels after being crosslinked.

Since the myocardium consists of muscular tissue in constant contraction and relaxation cycles, a cyclical compressive stress test was performed for the prepared hydrogels. Each test was composed of 50 compression and release cycles to detect if the hydrogels’ integrity was maintained after repetitive 30% strain compressions. As shown in Figure 2F–J, during the first cycle, all hydrogels reveal a slightly higher stress than in the following cycles. Nonetheless, the compressive stress is never higher than 4 kPa, which is in accordance with a previous study, where a gelatin methacrylate-oxidized dextran hydrogel, also designed for myocardial regeneration, never had its cyclical compressive stress going over 5 kPa.^15^ On the one hand, the hydrogel without LNFs breaks during the first cycle, and the following cycles do not look like each other. On the other hand, all hydrogels containing LNFs maintain their physical integrity, with very similar compression and release cycles measured on each sample, highlighting the mechanical reinforcement provided by the LNFs. This result contrasts with a previous study, where, in a similar test, a hydrogel of gelatin methacrylate slightly loses its integrity during the first 20 compression cycles before reaching stability.^15^ Additionally, the addition of AuNPs to the formulation did not affect the hydrogels’ compressive stress resilience.

### 3.2 Electrical Conductivity

**Figure 3:**
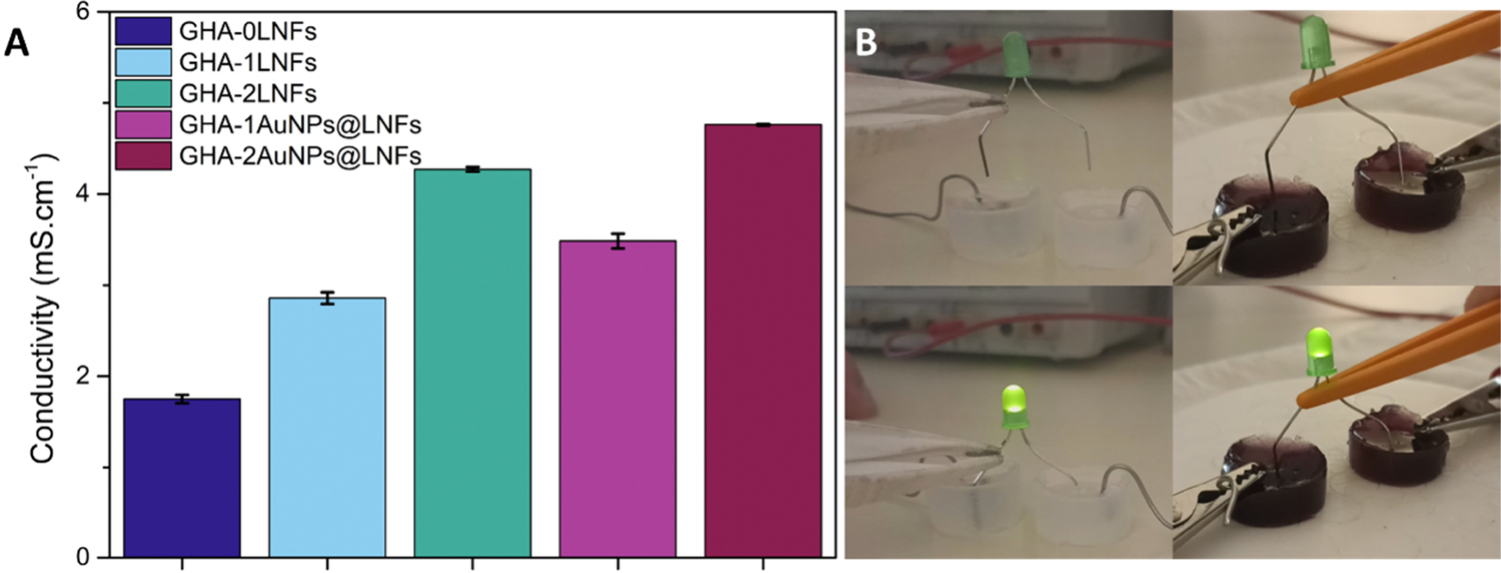
(A) Graphical display of the measured conductivity of all hydrogels at 37 °C. The error bars represent the SD (*n* = 3). (B) Photographs of GHA-2LNFs and GHA-2AuNPs@LNFs hydrogels (left and right, respectively) integrated into an electrical circuit before and after connecting a LED (top and bottom, respectively), allowing it to be lit.

Cardiomyocytes are electrically excitable cardiac cells that rapidly communicate between themselves through electrical impulses, resulting in a synchronized myocardial beating.^52^ So, when preparing a biomaterial for myocardium regeneration, its electrical conductivity must be considered to prevent arrhythmias.^53^ The electrical conductivity of all prepared hydrogels was measured to investigate the impact of the addition of LNFs, particularly AuNPs@LNFs hybrids. As expected, the conductivity of GHA-0LNFs was very low, under 2.0 mS cm^–1^ (Figure 3A), because none of the hydrogel components are deemed conductive. However, it is revealed that LNFs alone add enough electrical conductivity to the hydrogels (ca. 3–4 mS cm^–1^), matching the transversal and longitudinal conductivity values measured through a cardiomyocyte (0.6 and 2.1 mS cm^–1^, respectively).^52^ Most importantly, the GHA-2LNFs hydrogel has sufficient conductivity to match the one of the cytoplasm (3.0 mS cm^–1^).^52^ This is due to the LNF’s long fibrillar structure, the charged and aromatic groups present in lysozyme amino acid residues, and the densely packed concentration of hydrogen bonds.^54^ The functionalization of LNFs with AuNPs further improved the electrical conductivity of the corresponding hydrogels by around 0.5 mS cm^–1^. Although this increase of electrical conductivity is somewhat low, possibly far away from the percolation point, it means that, if needed, the incorporation of a higher quantity of AuNPs into the LNFs would further increase the obtained values.^55^ Nonetheless, as previously stated, this small amount of added AuNPs@LNFs hybrids into the formulation is adequate to boost the electrical conductivity to match the one of cytoplasm. As shown in Figure 3B, Video Clip S2, and Video Clip S3, when incorporated into an electrical circuit, this hydrogel allows a LED to be lit. Recent studies regarding conductive hydrogels for myocardium regeneration reported conductivities under 1.0 mS cm^–1^ for a gelatin methacrylate and oxidized dextran hydrogel,^15^ 0.4 mS cm^–1^ for a polyaniline and poly(ethylene glycol diacrylate)-based hydrogel,^53^ and 0.01 mS cm^–1^ for a functionalized HA and alginate hydrogel.^56^ Although in these works, the conductivity was not measured at 37 °C, which would increase the conductivity measurements,^57^ the much higher values obtained in the present work still highlight the achieved electroconductivity values.

### 3.3 Antioxidant Activity

**Figure 4:**
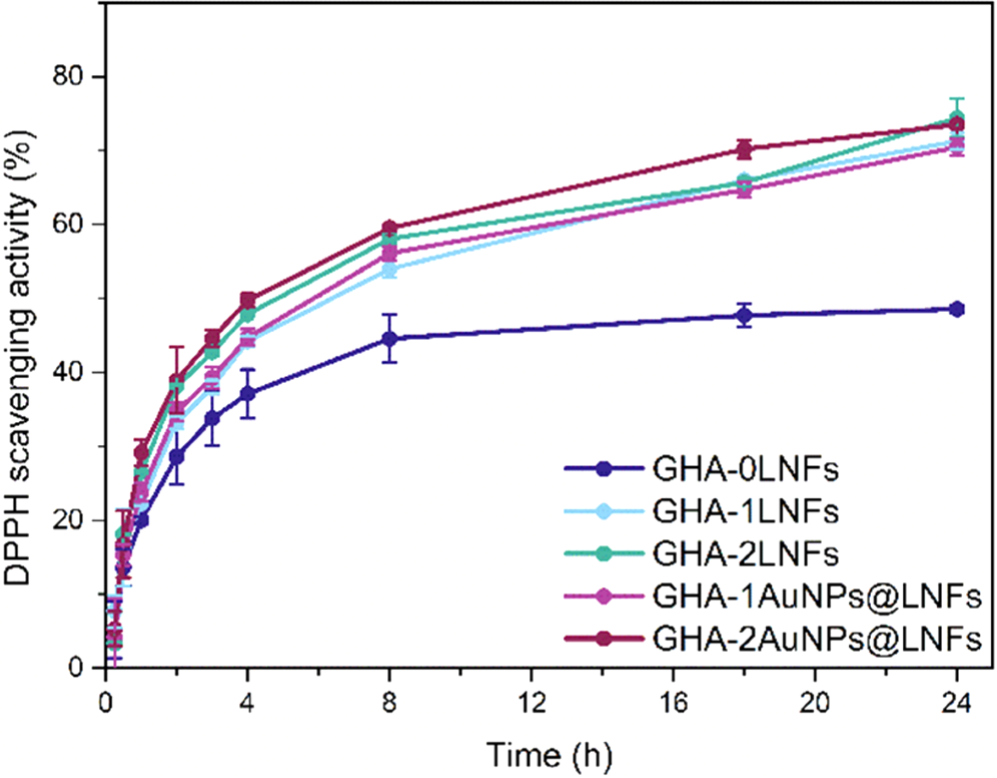
DPPH scavenging activity of the prepared injectable hydrogels. The error bars represent the SD (*n* = 3).

Antioxidants exist naturally in the organism to counter the resulting ROS production that originates from the normal functioning of the organs and tissues, maintaining the oxidative stress balance.^58^ When an injury occurs, such as a myocardial infarction, there is an inflammatory response, creating excess ROS that further damage the injured tissue.^26,58^ To restore the oxidative stress balance, additional antioxidants are needed in the affected area. One way to do that is to incorporate a biomaterial with antioxidative activity into the injured tissue.^26^ Due to the high content of amino acid residues with radical scavenging properties in egg white lysozyme sequence,^59^ even in its amyloid form, LNFs have demonstrated their value as antioxidant agents.^16,20^Figure 4 displays the antioxidant activity of the injectable hydrogels measured by the scavenging of DPPH for 24 h at 37 °C. All hydrogels show antioxidant activity; however, after 8 h, the scavenging activity of GHA-0LNFs plateaus at around 40%, while the others reached over 60% of activity, which continues to increase for the remaining time, attaining around 70% scavenging activity after 24 h. Therefore, by combining two biopolymers with antioxidant activity, namely, gelatin^60^ and HA,^61^ into a hydrogel, a DPPH scavenging activity of 40% is achieved, but adding a small quantity of LNFs, the antioxidant activity of the hydrogels is improved up to 30%. The antioxidant activity of gelatin also comes from the presence of amino acid residues with radical scavenging properties in its chain.^60^ The antioxidant activity of HA is due to its role in the activation and modulation of the inflammatory response, which includes scavenging activity against ROS.^61^ This improvement contrasts with the values obtained in a similar study about injectable electroconductive hydrogels of polyaniline emeraldine or sulfonated polyaniline.^53^ It revealed a DPPH scavenging activity of 60%, only 10–20% higher than the other formulations compared in the same study. Besides, 20% of scavenging activity was observable already at 1 h after incubation of the hydrogels containing LNFs. This rapid antioxidant activity might help prevent extended damage caused by ROS on the infarcted myocardium site.^62^ The addition of AuNPs did not affect the LNFs DPPH scavenging performance.

### 3.4 Swelling and Bioresorbability

**Figure 5:**
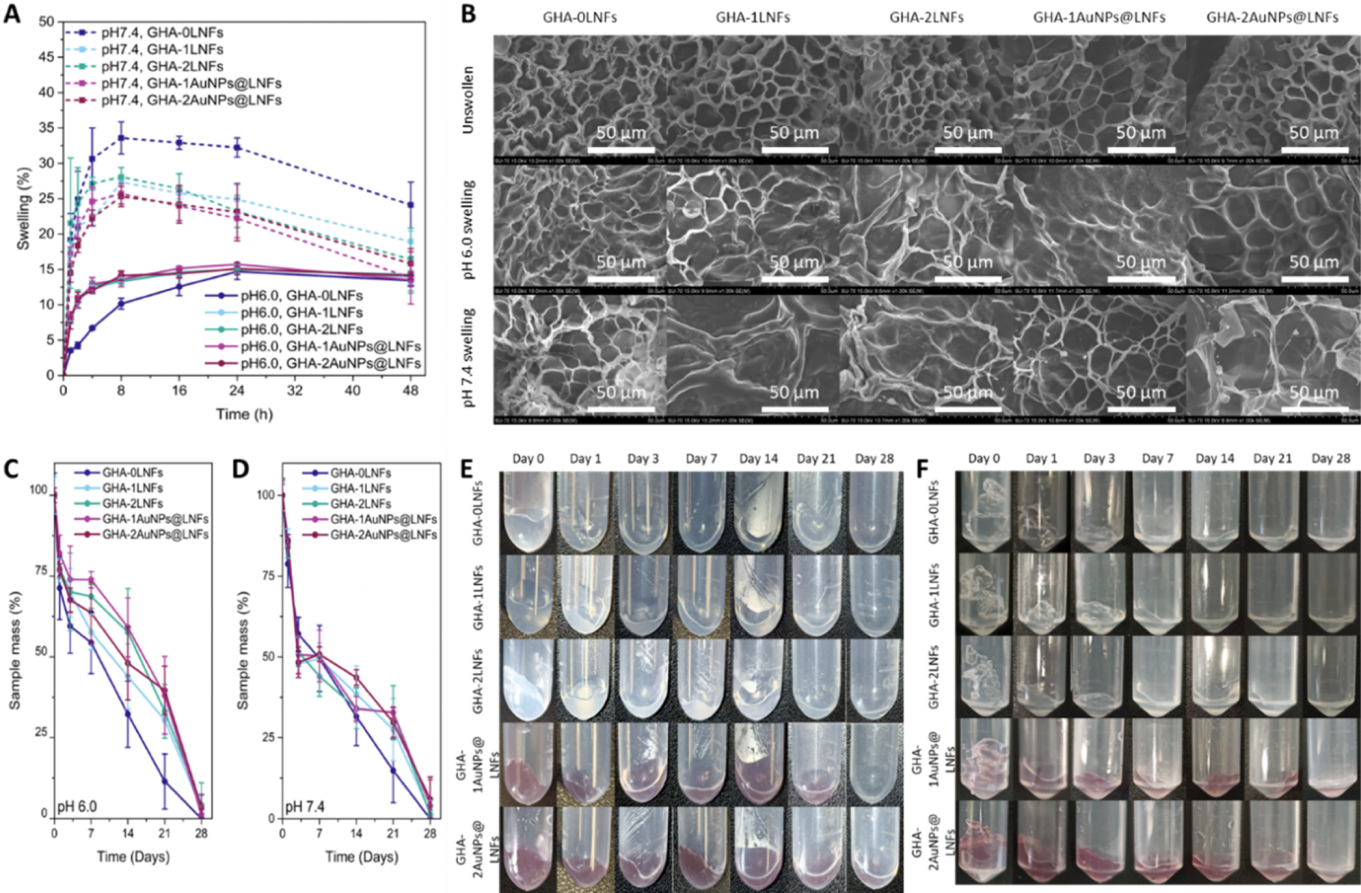
(A) Swelling rate of the hydrogels incubated with agitation of 100 rpm at 37 °C in PBS with pH 6.0 (solid lines) or in PBS with pH 7.4 (dotted lines). The error bars represent the SD (*n* = 3). (B) SEM micrographs of the different hydrogels (GHA-0LNFs, GHA-1LNFs, GHA-2LNFs, GHA-1AuNPs@LNFs, and GHA-2AuNPs@LNFs, respectively, from left to right columns) before swelling (top line) and after swelling at different pH values (pH 6.0 middle line or pH 7.4 bottom line). Scale bars are 50.0 μm. (C, D) Bioresorbability rate of the different injectable hydrogels incubated in PBS with pH 6.0 and pH 7.4, respectively. Percentages calculated through the percentage of mass decrease. The error bars represent the SD (*n* ≥ 4). (E–F) Photographs of the different hydrogels incubated in PBS with pH 6.0 and pH 7.4, respectively, at the different measured time points.

The swelling behavior of the hydrogels was determined by incubating them in PBS with pH 6.0 or 7.4 (representing the pH in inflamed and healthy tissues, respectively) in an orbital mixer at 100 rpm at 37 °C. Over a period of 48 h, the samples were weighed to calculate their swelling ratio. As shown in Figure 5A, at pH 6.0, the samples containing LNFs or AuNPs@LNFs reach a swelling equilibrium much faster than the ones without LNFs (4 h instead of 24 h). This phenomenon is due to the fact that the lysozyme isoelectric point is 11.35.^63^ At pH 6.0, lysozyme groups are protonated, resulting in positively charged nanofibers. It has been previously reported that hydrogels prepared from positively charged biopolymers, such as chitosan, at lower pH values have higher swelling ratios than at higher pH values.^64^ After reaching equilibrium, the swelling ratio is maintained at around 15% for all samples incubated in PBS with pH 6.0. For other hydrogels for the same application, reaching a swelling equilibrium might take 8 h for a gelatin methacrylate and oxidized dextran hydrogel^15^ or even 12 h for a chitosan-based hydrogel.^65^ On the one hand, to reach a faster equilibrium, this more rapid swelling observed on the samples containing LNFs or AuNPs@LNFs might allow a swifter release of components incorporated within these hydrogels (*e.g.*, drugs or growth factors).^64^ On the other hand, when incubated in PBS with pH 7.4, these hydrogels swell much faster, reaching around 32% (GHA-0LNFs hydrogel) and 27% swelling (hydrogels containing LNFs or AuNPs@LNFs) after 8 h of incubation. After that time, all hydrogels start losing mass (from material and/or water loss), reaching around 25% swelling (GHA-0LNFs hydrogel) and 22% swelling (hydrogels containing LNFs or AuNPs@LNFs) after 48 h of incubation, never reaching a swelling equilibrium. Additionally, to corroborate these results, the pore areas of each hydrogel were measured before and after incubation for 24 h. On average, the hydrogels incubated in PBS with pH 6.0 became 50% enlarged, while the ones incubated in PBS with 7.5 became 85% enlarged (Figure 5B).

Bioresorbability is a desired characteristic for materials used in regenerative medicine, since they must biodegrade as the new tissue is regenerated and formed. The bioresorbability of the prepared injectable hydrogels was determined by measuring their mass reduction when incubated either in PBS with pH 6.0 or pH 7.4 at 37 °C for 28 days. As shown in Figure 5C,D, all hydrogels were bioresorbable in both buffer solutions, being totally degraded in 28 days. The hydrogel without LNFs presented a similar degradation profile when incubated in either buffer. However, at pH 6.0, the hydrogels containing LNFs or AuNPs@LNFs revealed a slightly higher resistance to degradation during the first 2 weeks (under 50% degradation, Figure 5C). This behavior was not observed when incubated with PBS with pH 7.4, since the degradation profile was like the one observed for the hydrogel without LNFs (degradation over 50% after day 3, Figure 5D). The point of the hydrogels containing LNFs or AuNPs@LNFs being more impervious to degradation at pH 6.0 means that those hydrogels will perform better while the surrounding tissue is still inflamed. On the one hand, this phenomenon might be explained by the lower swelling ratio observed when incubated in PBS with pH 6.0, in combination with the measured storage modulus, which was higher for the hydrogels containing either LNFs or AuNPs@LNFs (Figure 2D). On the other hand, the higher swelling ratio observed for the hydrogels incubated in PBS with pH 7.4 (Figure 5A) might explain the higher bioresorbability rate observed during the first days when incubated in the same buffer because a swollen hydrogel facilitates the exchange of degradation byproducts for fresh buffer, while being more accessible to suffer further degradation from within. This is the opposite to what was observed with chitosan-based hydrogels, where the degradation was faster at lower pH values.^63^ Due to ischemia, an inflamed tissue has lower pH values, around pH 6.5–6.0,^66^ therefore, this is a desirable result because, during the first weeks, the hydrogels containing LNFs or AuNPs@LNFs are more resilient to degradation, maintaining their structure longer for a lasting therapeutic effect. Additionally, it was observed that the addition of AuNPs to the hydrogels did not have any statistical impact on their bioresorbability (Figure 5E,F).

### 3.5 In Vitro Cytotoxicity

**Figure 6:**
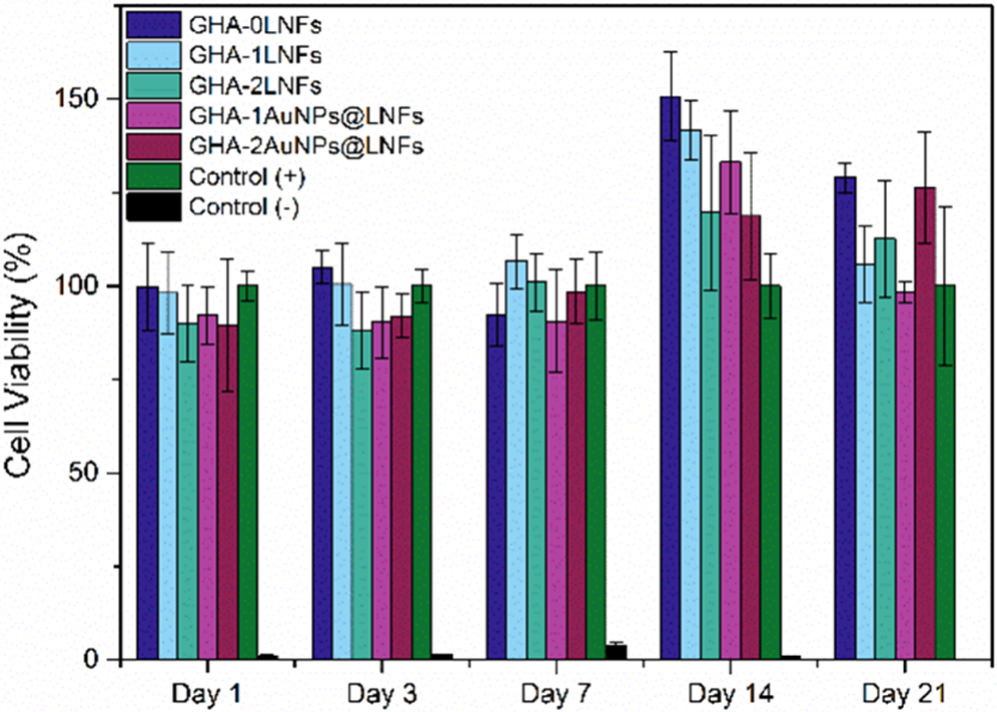
Cell viability and proliferation of H9c2 cells incubated with the injectable hydrogels for 1, 3, 7, 14, and 21 days. Cells incubated in a well without hydrogel (control (+)) and cells incubated without a hydrogel and later killed with Triton X-100 (control (−)). The results are expressed as mean ± SD. Compared to the controls (−), all samples have a statistically significant difference of cell viability (***p < 0.001) calculated through one-way ANOVA.

The cytotoxicity of the injectable hydrogels was assessed by measuring the cell viability and proliferation of H9c2 cells cultured with 25 μL of each hydrogel injected into each cultured well for 1, 3, 7, 14, and 21 days using the CellTiter-Glo assay.^32^ Positive (+) control groups were prepared for comparison by cultivating the same cells on wells without injecting any hydrogel. In contrast, the negative (−) control groups were prepared in the same fashion as the control (+), but the cells were killed with Triton X-100 a few hours prior to the cell viability measurement. As shown in Figure 6, on the first 7 days, the cell viability in all hydrogels was around 100%, like those of the control. However, on day 14, it was visible that the cells were thriving and proliferating when incubated in all hydrogels, reaching values of 125–150% of proliferation. On day 21, there was a relatively steep decrease of cell viability, but no statistically significant differences (one-way analysis of variance (ANOVA)) were observed when compared with the control, probably because there was no more space in the wells for further cell proliferation, or there were no more nutrients in the media for cell proliferation. Furthermore, these results also show that the addition of LNFs and AuNPs to the gelatin-HA injectable hydrogels did not change their cell viability, which indicates that their quantities can be adjusted to tune the other properties of the hydrogels, such as conductivity and bioresorbability ratio, without affecting the cell viability.

### 3.6 Ex Vivo Computer Tomography

**Figure 7:**
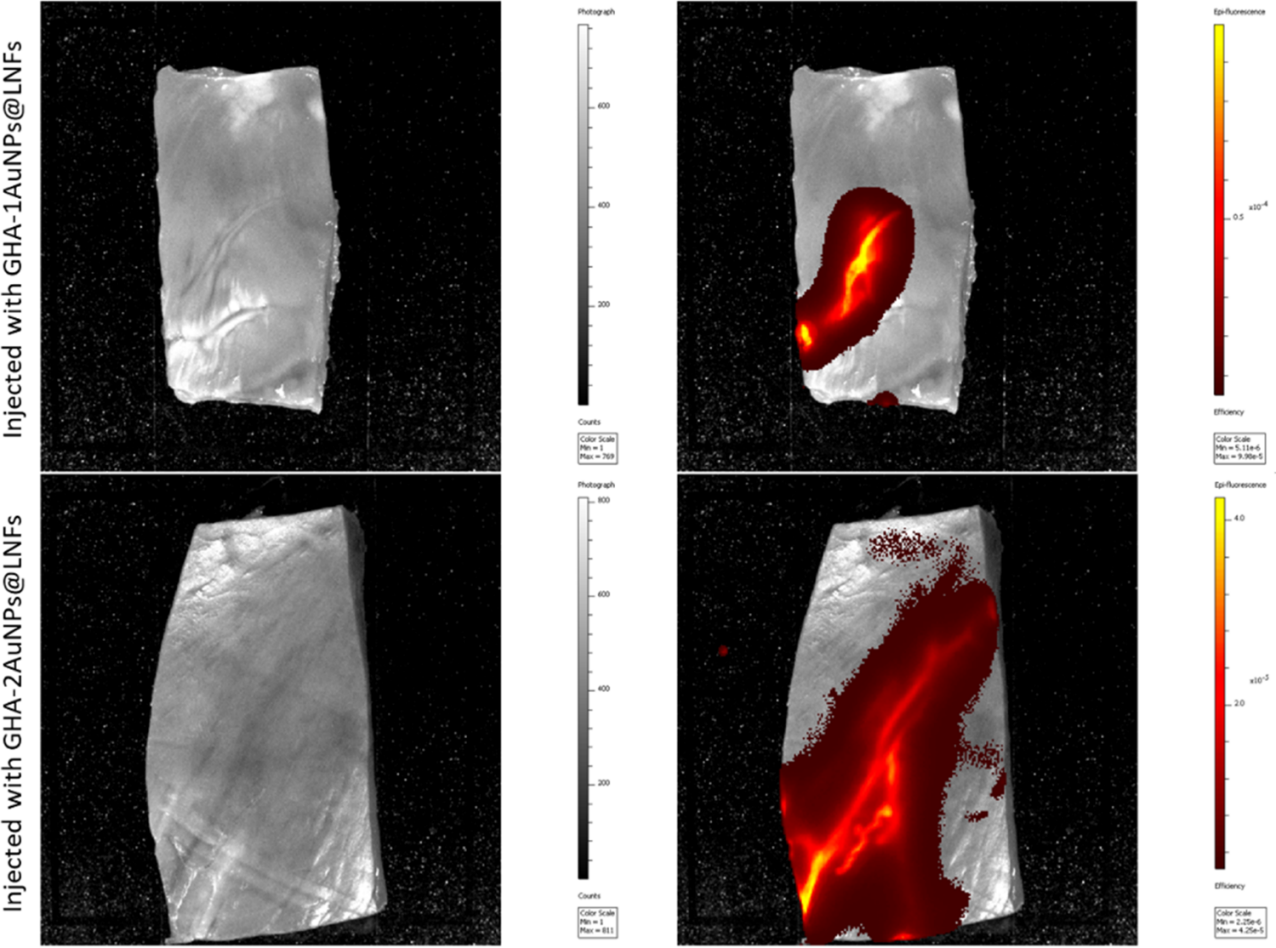
Myocardium pieces injected either with GHA-1AuNPs@LNFs or GHA-2AuNPs@LNFs (top and bottom, respectively). The images on the left were photographically obtained, while the images on the right were obtained through fluorescence, highlighting the presence of AuNPs.

Computer tomography was performed to noninvasively detect AuNPs inside myocardial tissue through fluorescence.^29^ In Figure 7 are shown two pieces of myocardium injected either with GHA-1AuNPs@LNFs or GHA-2AuNPs@LNFs (top and bottom, respectively). Due to the presence of AuNPs, the images obtained through fluorescence reveal a good contrast where the hydrogels were administrated. Furthermore, both hydrogel formulations were able to spread through the myocardial tissue and did not settle in the administration spot. This leads to a higher contact area with the damaged tissue, increasing the therapeutic effect. All in all, the presence of AuNPs in the hydrogel formulation allows its detection to confirm a good administration, as well as the possibility of monitoring hydrogel degradation and AuNPs excretion.^29^

### 3.7 Drug Loading and Release

**Figure 8:**
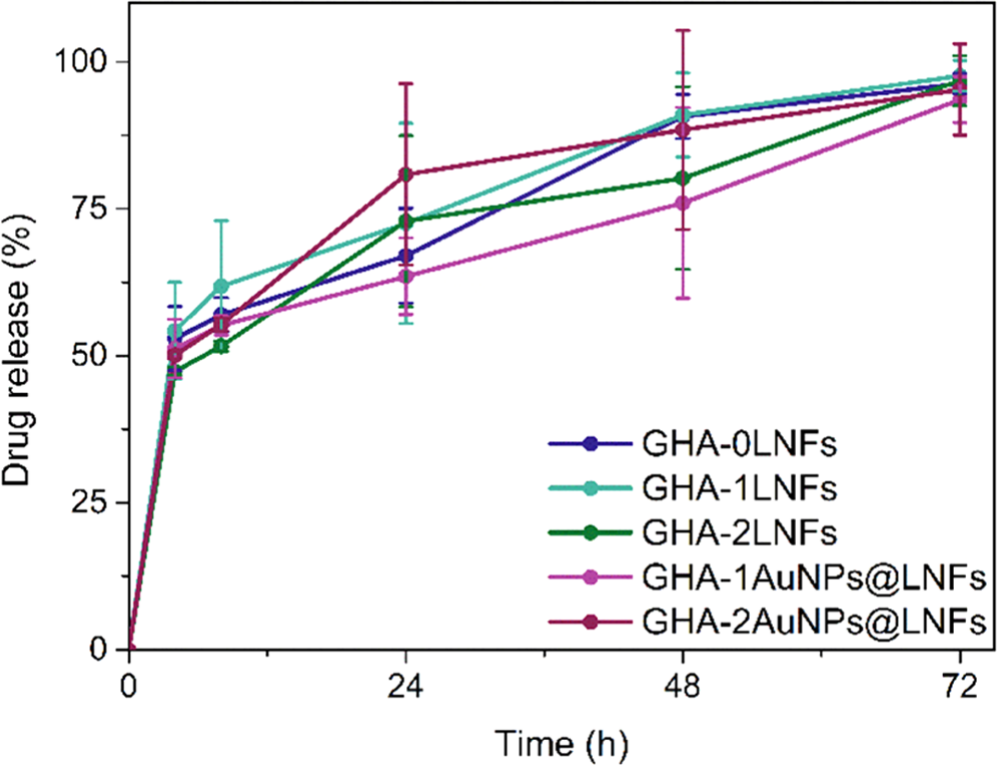
Drug release profiles (%) of the incorporated drug during incubation of each hydrogel on PBS with pH 6.0 at 37 °C, with an agitation of 100 rpm. The error bars represent the SD (*n* = 3).

Since the importance of hydrogels is also manifested in their ability to be able to deliver drugs to the heart,^4^ injecting hydrogels previously loaded with drug molecules is a simple strategy to administrate them without having to surpass all natural barriers of the organism.^67^ The used model drug was CHIR 99021 trihydrochloride, a hydrophilic version of CHIR 99021, which is a glycogen synthase kinase 3 inhibitor,^39^ which also initiates cardiomyocyte differentiation^68^ and proliferation.^39^ Due to its hydrophilic nature, incorporating 2.0 mg mL^–1^ of this model drug into the hydrogels was a straightforward task. As shown in Figure 8, in the first 4 h, 50% of the incorporated drug was burst-released from all hydrogels. From that time point on, the rest of the incorporated drug was continuously released at a slower rate. After 72 h of incubation, the incorporated drug was totally released from within all hydrogels. The presence of LNFs or AuNPs did not affect the release profiles. The observed burst release of the model drug is extremely appropriate in the first hours post hydrogel injection, allowing a therapeutic effect from day 0.^68^ Since all remaining incorporated drug is slowly released in the following hours, the therapeutic effect is maintained throughout that period. Furthermore, these hydrogels allowed this hydrophilic drug to be released faster than other hydrogels reported in the literature. For instance, a peptide-based injectable hydrogel released 80% of the hydrophilic drug contained within (Salvianolic acid B), only after 96 h of incubation.^69^ And, a ROS-sensitive crosslinked poly(vinyl alcohol) hydrogel, after 8 days, only released 50% of a very water soluble growth factor.^70^

## 4 Conclusions

In this study, biopolymeric injectable hydrogels for myocardial regeneration applications were developed. These hydrogels contained gelatin, HA, different amounts of LNFs, and in two cases, AuNPs embedded into the LNFs. The in situ preparation of AuNPs within the LNFs resulted in AuNPs@LNFs hybrids and showed to be a valuable step to avoid AuNPs aggregation. The addition of LNFs or AuNPs@LNFs to these hydrogels did not affect their injectability, biocompatibility toward H9c2 cells, or drug release performance. In contrast, the incorporation of LNFs into the hydrogels improved their rheological properties, mechanical stress resilience, electrical conductivity, antioxidant activity, and added desired pH-responsive bioresorbability and swelling. Additionally, the presence of AuNPs in the formulation further increased the hydrogels’ electrical conductivity, making them monitorable through computer tomography. All considered, these results highlight the resourcefulness of LNFs to improve biopolymeric hydrogels regarding different properties and functionalities of interest for myocardial regeneration applications.

## Supporting Information Available

Hydrogel manual injectability (Video Clip S1) (MP4)
Conductive hydrogel containing LNFs (Video Clip S2) (MP4)
Conductive hydrogel containing AuNPs@LNFs (Video Clip S3) (MP4)

## Abbreviations

ANOVA: analysis of variance
ATR-FTIR: attenuated total reflection-Fourier transform infrared
AuNPs: gold nanoparticles
DES: deep eutectic solvent
DMEM: Dulbecco’s modified Eagle’s medium
DPPH: 2,2-diphenyl-1-picrylhydrazyl
ECM: extracellular matrix
G: gauge
H9c2: rat cardiomyoblast
HA: hyaluronic acid
HIFBS: heat-inactivated fetal bovine serum
HPLC–DAD: high-performance liquid chromatography-diode-array detection
LNFs: lysozyme nanofibrils
NEAA: nonessential amino acids
NFs: protein nanofibrils
PBS: phosphate-buffered saline
PEGDGE: poly(ethylene glycol) diglycidyl ether
ROS: reactive oxygen species
SD: standard deviation
SEM: scanning electron microscopy
